# Immune remodeling during cervical cancer chemoradiotherapy: temporal biomarkers and the timing of checkpoint blockade

**DOI:** 10.3389/fimmu.2026.1947595

**Published:** 2026-09-03

**Authors:** Chenguang Yang, Xuan Song, Hongmei Sun, Min Chen, Xialing Zhu

**Affiliations:** 1 Department of Gynaecology and Obstetrics, Affiliated Xuancheng Hospital of Wannan Medical University, Xuancheng, Anhui, China; 2 Department of General Medicine, Affiliated Xuancheng Hospital of Wannan Medical University, Xuancheng, Anhui, China

**Keywords:** cervical cancer, chemoradiotherapy, circulating tumor HPV DNA, immune checkpoint inhibitor, radiation-induced lymphopenia, temporal biomarkers, tumor immune microenvironment, type I interferon

## Abstract

Concurrent chemoradiotherapy (CRT) is the backbone of curative treatment for locally advanced cervical cancer, and PD-(L)1 blockade has entered first-line practice. Yet the pivotal trials diverge: pembrolizumab added to CRT improved survival in KEYNOTE-A18, whereas durvalumab did not in CALLA. Most prior syntheses organize the cervical tumor immune microenvironment by cell type and rely on cross-sectional, pre-treatment sampling, and several predate KEYNOTE-A18 and recent longitudinal multi-omic data. Here we reframe the question along the treatment time axis. Section 1.1 reports that evidence base in full, under tiered eligibility criteria. Drawing on the still-small body of studies with paired or serial pre-, on-, and post-treatment sampling of tumor and blood, we reconstruct four sequential states: an HPV-shaped baseline ecology; an early immunogenic priming window driven by DNA-damage sensing and type I interferon; a mid-treatment window in which intratumoral activation coexists with radiation-induced lymphodepletion and compensatory checkpoint upregulation; and a post-treatment state of viral-antigen persistence, minimal residual disease, and adaptive resistance. We use this lens to interpret CRT–immune checkpoint inhibitor (ICI) trial heterogeneity without over-attributing it, and derive a falsifiable prediction, which neither pivotal trial can adjudicate and which the best available randomized-evidence synthesis currently opposes: blockade delivered within the priming window may outperform equal exposure given as post-CRT maintenance, in patients who mount on-treatment activation without catastrophic lymphodepletion. This is a mechanistically motivated hypothesis, not an inference from existing results. Testing needs a factorial or biomarker-stratified trial. We also propose a temporal biomarker framework spanning baseline-predictive, on-treatment early-response, and post-treatment residual-disease markers, graded by evidence and offered as a research agenda. For each stage we name the design that would qualify or refute it. Stage 1 needs registered analysis of archival trial tissue. Stage 2 needs a nested correlative sub-study with prespecified effect modifiers. Stage 3 needs a blinded, prespecified prognostic cohort. We propose, as a hypothesis for prospective testing, that response to CRT–ICI may depend less on any single baseline feature than on whether a patient completes the dynamic transition from viral-antigen exposure through antigen presentation and T-cell recruitment to durable surveillance.

## 1 Introduction

Cervical cancer remains a leading cause of cancer death in women worldwide, and a large proportion of patients present with locally advanced disease (LACC) for which concurrent cisplatin-based chemoradiotherapy (CRT) is the standard of care. Because nearly all cervical cancers are driven by persistent high-risk human papillomavirus (HPV) infection, the disease is, at least in principle, an attractive setting for immunotherapy: the tumors express foreign viral oncoproteins, and radiotherapy is itself a potent modulator of anti-tumor immunity. This rationale has now been tested at scale. In the persistent, recurrent, or metastatic setting, PD-1 blockade produced durable responses and a survival benefit, establishing pembrolizumab and cemiplimab as active agents (1–3), and PD-1 blockade combined with chemotherapy became first-line therapy (1). The decisive step for curative-intent disease came with KEYNOTE-A18, in which pembrolizumab added to CRT and continued as maintenance improved progression-free and overall survival in high-risk LACC (4, 5).

The field, however, confronts an instructive contradiction. In CALLA, durvalumab (anti-PD-L1) concurrent with and following CRT did not significantly improve progression-free survival (6). Two large, well-conducted trials of PD-(L)1 blockade added to the same chemoradiotherapy backbone thus reached opposite conclusions. The temptation is to explain this divergence by a single mechanism, whether antibody target, PD-L1 status, or drug potency. But the trials differed simultaneously in drug class, schedule, patient risk profile, and statistical design, and the biological readouts that would adjudicate between explanations were largely not collected serially.

Most existing reviews of the cervical tumor immune microenvironment (TIME) are organized by immune cell lineage and lean on baseline, cross-sectional data. The most comprehensive systematic synthesis of immune correlates in CRT-treated cervical cancer was assembled before the KEYNOTE-A18 era (7), and even recent narrative and mechanistic reviews of cervical cancer immunotherapy remain structured around mechanism or cell type rather than time (8). The most direct point of comparison is a 2026 synthesis of radiotherapy–immunotherapy reprogramming of the cervical TIME (9), which surveys the mechanistic synergy comprehensively but still organizes it by pathway and cell type rather than by treatment phase. Our contribution differs in three specific ways. We organize the evidence along the treatment time axis rather than by mechanism. We restrict the core evidence base to studies with paired or serial sampling, excluding cross-sectional snapshots from the primary argument. And we distill the trajectory into an explicit, testable temporal biomarker framework. This matters because CRT is an active, weeks-long perturbation that remodels both tumor and host immunity, so a pre-treatment biopsy captures only the starting state of a moving system.

The field is already moving toward this time-resolved reading. Longitudinal multi-omic profiling now shows CRT concurrently remodeling tumor-cell and immune compartments, and a phase II trial has defined spatiotemporal immune determinants of response to immune-checkpoint re-challenge in advanced cervical cancer (10). The value a review adds is to integrate scattered longitudinal observations into a single operational framework with biomarker stratification, which no individual primary study provides.

**Figure 1:**
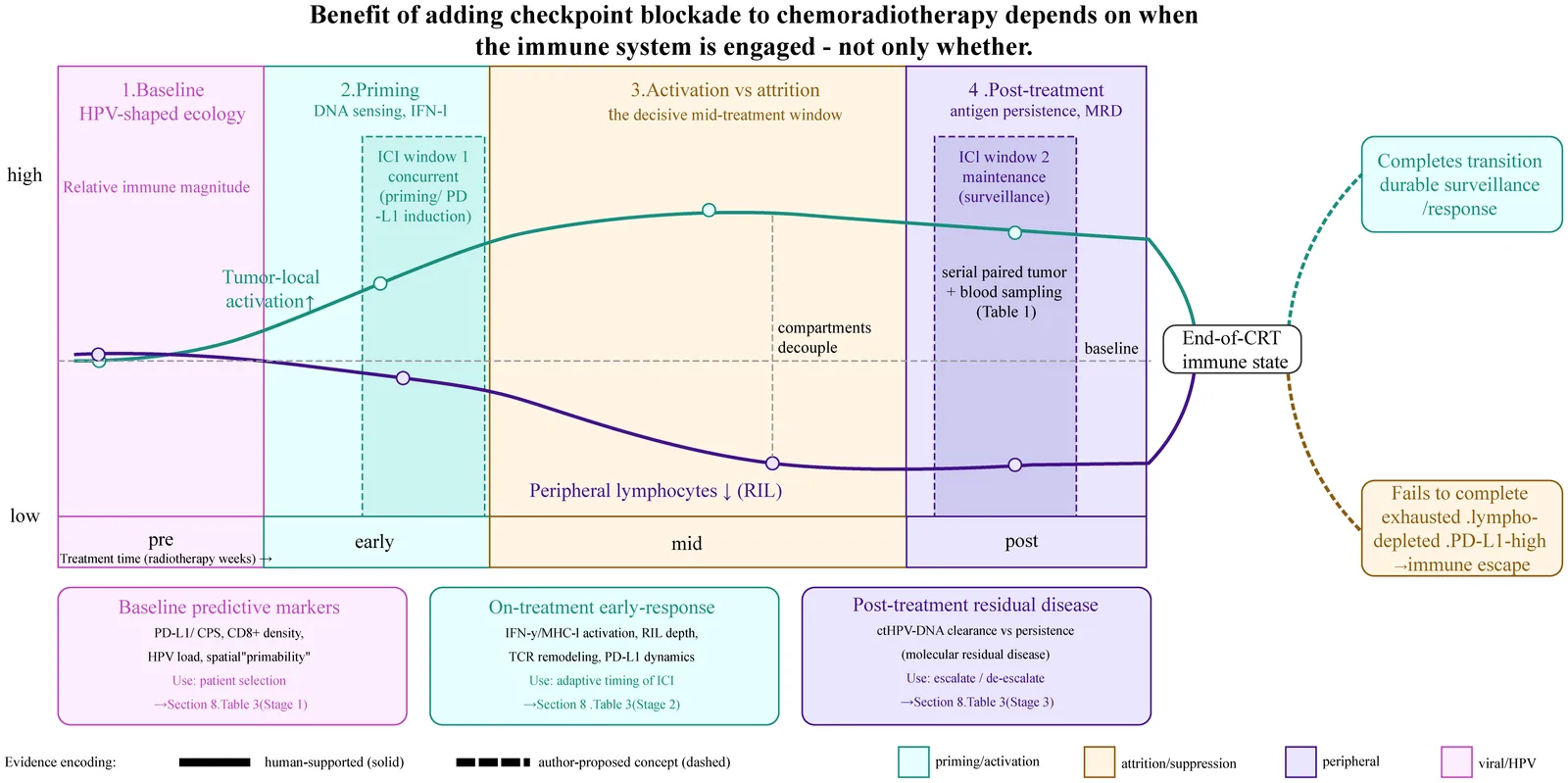
Immune remodeling across cervical chemoradiotherapy: a four-state trajectory and the timing of checkpoint blockade. A left-to-right treatment-week timeline with two parallel lanes (tumor-local, upper; peripheral blood, lower) plotted against a shared vertical axis of relative immune magnitude (normalized to baseline). The two trajectories diverge through the mid-treatment window, where intratumoral activation rises while circulating lymphocytes decline. Open circles on each lane mark serial paired tumor/blood sampling timepoints (pre, early, mid, post; **Table 1**). Four state bands of unequal width (baseline, HPV-shaped; priming; activation-versus-attrition, the widest; post-treatment) are overlaid with two dashed “ICI timing window” zones, labeled concurrent (priming/PD-L1 induction) and maintenance (surveillance). The timeline starts at the first fraction of chemoradiation, so induction (pre-CRT) dosing is outside its span; that strategy is treated in Section 2.1 and would occupy a further window to the left of the concurrent zone. At the right, both lanes converge into a single end-of-CRT immune-state node, from which a terminal fork diverges into a favorable branch (completes the transition → durable surveillance/response) and an unfavorable branch (fails to complete → exhausted, lymphodepleted, PD-L1-high → immune escape). Three caption notes map the baseline, on-treatment, and post-treatment biomarker tiers to the framework of Section 8 and **Table 3**. Solid elements denote human-supported features (the trajectories and their convergence); dashed elements (the ICI windows and the outcome fork) denote the author-proposed conceptual layer, advanced as a hypothesis for prospective testing rather than as an established schedule effect (Section 7). Color semantics: teal, priming/activation; amber, attrition/suppression; blue, peripheral; purple, viral/HPV. This image also serves as the graphical abstract. CRT, chemoradiotherapy; ICI, immune checkpoint inhibitor; PD-L1, programmed death-ligand 1; HPV, human papillomavirus; CPS, combined positive score; MHC-I, major histocompatibility complex class I; IFN-γ, interferon-gamma; RIL, radiation-induced lymphopenia; TCR, T-cell receptor; ctHPV-DNA, circulating tumor HPV DNA; MRD, molecular residual disease.

This review adopts a temporal organizing principle throughout. Drawing on the still-limited set of paired- and serial-sampling studies, we read tumor-local and peripheral signals together without equating them. We reconstruct the immune trajectory as four sequential states (baseline → priming → activation-versus-attrition → persistence/adaptive resistance) (**Figure 1**) and integrate spatial and single-cell evidence. We then use the trajectory to interpret trial heterogeneity, distinguishing demonstrated associations from mechanistic inference. This yields two deliverables that a cell-type-organized synthesis does not. One is a testable prediction about the timing of blockade relative to radiation (Section 7). The other is a temporal biomarker framework, graded by strength of evidence (Section 8). Where we make a prediction, we name the design that would refute it. The central claim is a hypothesis, not a conclusion the existing trials license: the benefit of adding ICI to CRT may depend on when the immune system is engaged. That timing has been underweighted relative to the simpler question of whether to combine the two at all.

### 1.1 Evidence base: literature search strategy and study selection

This is a narrative review, not a systematic one. We therefore report its evidence base in full, so that a reader can trace the synthesis and audit the selection rules, following quality standards proposed for narrative reviews (11). We searched PubMed/MEDLINE, Embase, Web of Science Core Collection and the Cochrane Library from 1 January 2000 to 10 July 2026. ClinicalTrials.gov supplied registered and reported trials. We also hand-searched the reference lists of retrieved articles and of the major prior syntheses in this area (7–9). The search combined three concept blocks with the Boolean operator AND. Block (i), disease: “cervical cancer” OR “cervical carcinoma” OR “cervical neoplasms” OR “Uterine Cervical Neoplasms”[MeSH] OR “endocervical adenocarcinoma”. Block (ii), treatment: “chemoradiotherapy” OR “chemoradiation” OR “radiotherapy” OR “radiation” OR “brachytherapy” OR “cisplatin” OR “immune checkpoint inhibitor” OR “PD-1” OR “PD-L1” OR “pembrolizumab” OR “durvalumab” OR “nivolumab”. Block (iii), immune process or readout: “tumor immune microenvironment” OR “tumor-infiltrating lymphocyte” OR “type I interferon” OR “cGAS” OR “STING” OR “MHC class I” OR “lymphopenia” OR “T-cell receptor repertoire” OR “circulating tumor DNA” OR “HPV DNA” OR “single-cell” OR “spatial transcriptomics” OR “tertiary lymphoid structure” OR “therapeutic vaccine” OR “adoptive cell therapy”. Terms were mapped to MeSH headings where available and run as title/abstract free text otherwise. No language filter was applied at the search stage. Records without an English-language full text or abstract were excluded at screening. The search was last re-run on 10 July 2026.

Two authors (C.Y., X.S.) screened records on title and abstract and then in full text. Disagreements were resolved by discussion, and where unresolved by a third author. Because the organizing claim of this review is temporal, we applied a tiered eligibility scheme instead of a single inclusion threshold. The tier assigned to a study sets the weight it carries in the argument. Tier 1 (core evidence; **Table 1**) required human studies in cervical cancer with paired or serial sampling of tumor or blood at two or more timepoints spanning pre-, on-, or post-treatment, reporting an immune, T-cell-receptor-repertoire, or circulating tumor DNA (ctDNA)/circulating tumor HPV DNA (ctHPV-DNA) readout. These studies alone support the trajectory reconstructed in Sections 3–6. Tier 2 comprised randomized or prospective clinical trials of PD-(L)1 blockade combined with, or sequenced around, chemoradiotherapy in cervical cancer, with or without correlative sampling (**Table 2**). Tier 3 comprised mechanistic, preclinical, cross-sectional human, or non-cervical clinical studies. We admitted a Tier 3 source only where it supplies a mechanism or principle that no cervical longitudinal dataset yet provides. Every Tier 3 citation used to support an inference about cervical cancer is identified as extrapolated in the running text, and graded C or D in **Tables 1** and **3**. The same convention governs the running text, not only the citations: we state a claim resting on human cervical material without qualification. Where a claim is imported from another tumor type or from preclinical work, we label it extrapolated at the point of use, and grade it C or D, the levels used in **Tables 1** and **3**.

**Table 1:** Longitudinal human cervical studies with paired or serial pre-, on-, or post-treatment sampling.

Study (first author, year)	Design	n/sample type	Timepoints	Assay/omics	Key dynamic finding	Evidence level
Dorta-Estremera 2018 (43)	Prospective	20 tumor brushings/9 paired PBMC	Baseline + weeks 1/3/5	Flow cytometry/immune profiling	On-treatment intratumoral immune activation during CRT	A
Li 2021 (44)	Single-center prospective trial	55; tumor + PBMC	Before/during/after CRT	Lymphocyte subsets, PD-1/PD-L1, TCR	Coupled local–systemic dynamics; tumor PD-L1 78.1%→49.8%	A
Manzar 2024 (46)	Prospective multi-institutional	13; cervix swabs	Baseline/during CRT/post-brachytherapy	TCR-β deep sequencing (immunoSEQ)	HPV E6/E7-responsive clones expand on treatment; non-remodelers recurred	A
Cosper 2020 (45)	Prospective	115 biopsied pre- and 3 wk into CRT (20 paired mid-treatment)	Baseline + week 3	Bulk RNA-seq + HPV expression	Decreased local immune response; retained HPV in resistant tumors	A
Dai 2024 (74)	Paired scRNA-seq	Pre/post-CRT tumor	Pre and post	Single-cell RNA-seq	CRT-induced ACKR2^+^ tumor cells drive CD8 senescence	A
Cabel 2021 (64)	Mixed cohort (41 retrospective + 14 prospective)	55 HPV^+^ LACC; serial plasma	Serial during/after	Plasma ctHPV-DNA	ctDNA clearance tracks disease-free survival	A–B
Mayadev 2025 (CALLA ctDNA) (71)	Within-RCT correlative	185 baseline; plasma	Baseline/post-CRT/3-mo	Tumor-informed ultrasensitive ctDNA	Molecular residual disease predicts relapse/OS	A
Lan 2026 (10)	Phase II re-challenge trial	30; tumor	Serial + spatial	Spatial profiling	Spatiotemporal determinants of response to ICI re-challenge	A
Ray-Coquard 2026, COLIBRI (27)	Phase II window-of-opportunity trial	40; paired tumor biopsies	Baseline and after 1 cycle of induction ICI (pre-CRT)	Multiplex immunofluorescence; 27-gene HOT signature	Induction anti-PD-1 + anti-CTLA-4 raises intratumoral CD8+/FOXP3+ ratio and proliferative CD8+ density before CRT	A

Evidence base for Sections 2–6. Evidence-level categories (used across **Tables 1**, **3**): (A) human longitudinal direct, meaning same-patient paired/serial cervical sampling; (B) human cross-sectional/associative; (C) cross-cancer or preclinical extrapolation; (D) conceptual inference.

Single-timepoint, cross-sectional immune-ecosystem maps [Cao 2023 (81); Fan 2023 (82)] are cited in Section 2 for architectural reference but are not treatment-serial and are therefore not listed as longitudinal rows.

Section 3 specifies claim by claim, which mechanistic links in the priming model have been validated in human cervical cells, tissue or patients and which are extrapolated from non-cervical systems; **Table 3** grades the resulting biomarker candidates on the same basis (evidence levels A–D).

**Table 2:** CRT–ICI and key cervical immunotherapy trials.

Trial	Agent (target)	Setting/population	Schedule relative to CRT	Primary outcome	Serial/immune correlates
KEYNOTE-A18 (4, 5)	Pembrolizumab (anti-PD-1)	Curative-intent, high-risk LACC	Concurrent with CRT + maintenance	PFS and OS improved (positive)	Limited serial biology collected
CALLA (6)	Durvalumab (anti-PD-L1)	Curative-intent LACC	Concurrent with + following CRT	PFS not significantly improved (negative)	Within-trial tumor-informed ctDNA/MRD (71)
NICOL (93)	Nivolumab (anti-PD-1)	LACC	Concurrent with CRT	Phase 1 safety/feasibility	Serial tumor + blood sampling (combination-CRT)
COLIBRI (27)	Nivolumab + ipilimumab (anti-PD-1 + anti-CTLA-4)	Curative-intent LACC (squamous); phase II, single-arm	Induction (1 cycle) before CRT, then CRT, then nivolumab maintenance	Intratumoral CD8+/FOXP3+ ratio increased (primary endpoint met)	Paired pre-/post-induction tumor multiplex IF + gene signature
Duska 2025 (100)	Pembrolizumab (anti-PD-1)	Curative-intent LACC; randomized phase II	Concurrent with CRT vs sequential after CRT (no maintenance)	Safety/feasibility met; PFS and OS not different between schedules	Serial blood and tissue collected (translational)

Curative-intent CRT–ICI trials (Section 7).

For context, ICI regimens not combined with radiotherapy (first-line and recurrent/metastatic cervical cancer: KEYNOTE-826, cemiplimab, CheckMate 358, NRG-GY002) are discussed in Section 7 (1–3, 117, 118); they are omitted from this table to keep the focus on curative-intent CRT–ICI.

**Table 3:** The temporal biomarker framework at a glance (author-proposed).

Stage	Candidate marker	Sample	Evidence level	Intended clinical use
Baseline (predictive)	PD-L1/CPS	Tumor	B	Patient selection/baseline stratification
Baseline (predictive)	CD8^+^ infiltrate; inflamed/excluded/desert phenotype	Tumor	B	Identify tumors likely to be “primable” by CRT
On-treatment (early response)	IFN-γ/MHC-I activation signature	Tumor	C (extrapolated; A pending cervical validation)	Confirm priming; guide timing of ICI
On-treatment (early response)	Radiation-induced lymphopenia (RIL) depth	Blood	A–B	Flag attrition risk; motivate marrow-sparing RT
On-treatment (early response)	Tumor PD-L1/checkpoint dynamics	Tumor	B	Detect compensatory checkpoint upregulation
Post-treatment (residual disease)	ctHPV-DNA/tumor-informed ctDNA clearance	Blood	A (cervical-specific; within-RCT MRD from CALLA)	Detect MRD; adaptive escalation/de-escalation
Cross-stage (integrative)	Composite “primability” score	Tumor + blood	D (conceptual)	Trial-level stratification hypothesis
On-treatment (early response)	Intratumoral ICI target occupancy/drug exposure under lymphodepletion	Tumor + blood	D (conceptual; never measured in cervical CRT–ICI)	Test whether fixed-dose blockade achieves adequate occupancy when the lymphoid sink is depleted (Section 4)
Post-treatment (residual disease)	Tumor-informed, non-viral ctDNA for HPV-independent histologies	Blood	C (extrapolated; requires re-derivation)	Extend Stage 3 to HPV-independent disease, where ctHPV-DNA is undefined (Section 5.1)

Evidence levels as defined for **Table 1**; the evidence-level column makes the framework’s provisional status auditable (Section 8).

We excluded case reports, conference abstracts without a peer-reviewed full text, editorials and commentaries, studies confined to cervical intraepithelial neoplasia or to prophylactic (non-therapeutic) HPV vaccination, and purely dosimetric or radiobiological analyses without an immune endpoint. Review articles were not counted as primary evidence. We used them to identify primary sources, and to position the present synthesis against existing ones. Because this is a narrative synthesis, we did not construct a PRISMA flow diagram, and we make no claim of exhaustiveness for the Tier 3 mechanistic literature. The two tiers that carry the argument are instead enumerated completely. All eight qualifying Tier 1 studies are listed in **Table 1** and the Tier 2 trial set in **Table 2**, so that a reader can audit the core evidence base directly. This process has two limits. Tiered narrative selection does not remove selection bias, as a full systematic review with duplicate independent extraction and formal risk-of-bias appraisal would; the Tier 3 citations in particular reflect authorial judgment about mechanistic relevance. The Tier 1 yield is also small, and mixed in platform, sampling site and timepoint definition, which limits any quantitative synthesis. That scarcity is itself one of this review’s principal findings.

## 2 Baseline: the HPV-shaped immune ecology

**Figure 2:**
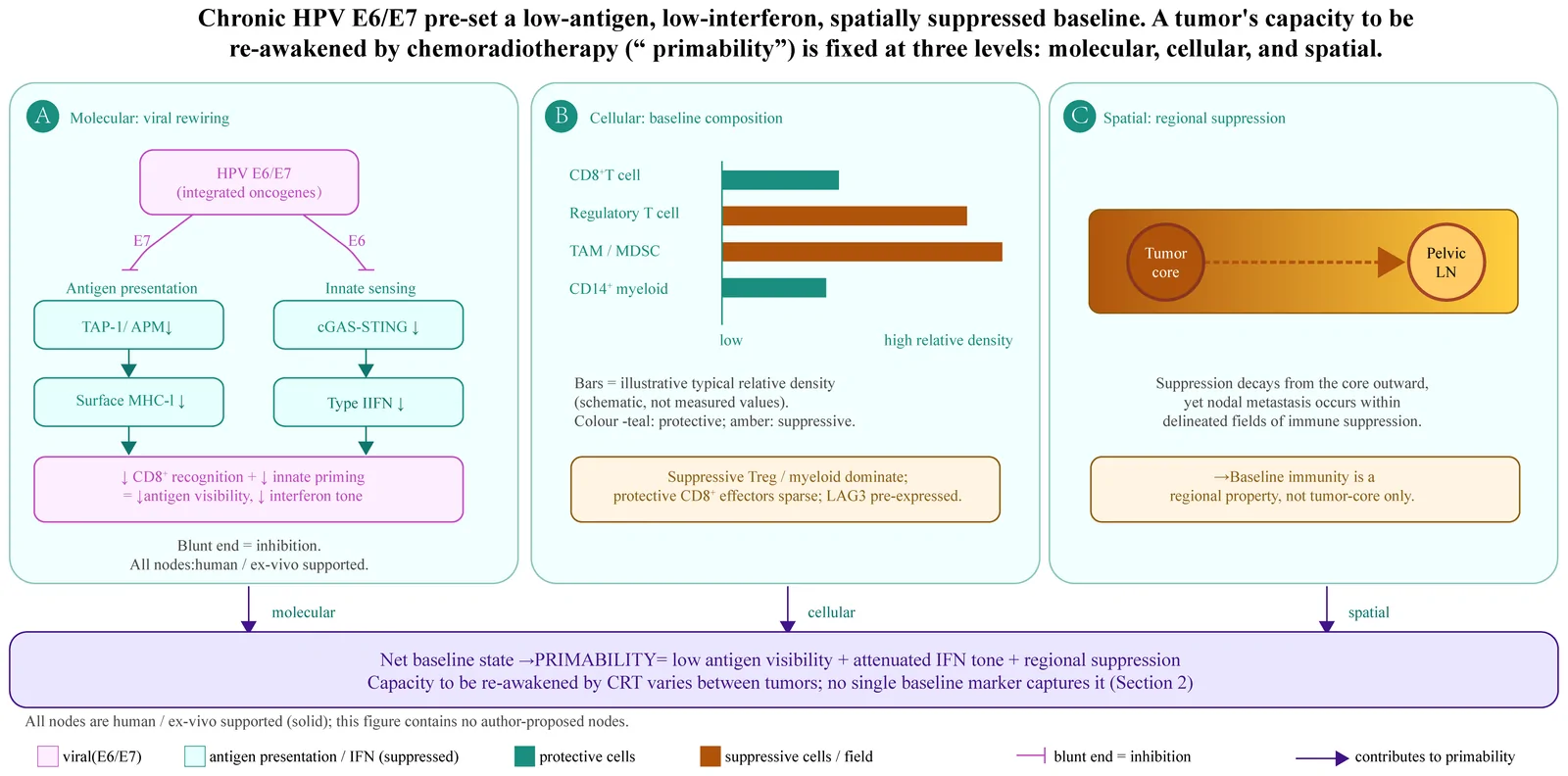
The HPV-shaped baseline sets each tumor’s primability at three levels. **(A)** Molecular: within the keratinocyte, integrated HPV E6/E7 downregulate TAP-1 and surface MHC class I (via E7) and impair the cGAS–STING/type I interferon axis (via E6) (blunt-ended connectors denote inhibition), yielding a pre-therapy tissue selected for reduced antigen visibility and attenuated interferon tone. **(B)** Cellular: composition of the baseline tumor immune microenvironment. Bar lengths show illustrative typical relative densities (schematic, not measured values). Color encodes function. Teal marks protective populations: CD8^+^ T cells, prognostic when high, and a benign CD14^+^ myeloid subset. Amber marks suppressive populations: regulatory T cells and tumor-associated macrophages/MDSC. Inhibitory receptors such as LAG3 are already expressed before therapy. **(C)** Spatial: a regional “field of immune suppression” decaying from the tumor core toward the pelvic lymph node, showing that baseline immunity is a regional property. Directional arrows show the three levels (molecular, cellular, spatial) converging on the net baseline “primability” readout. All nodes shown are supported by human or ex-vivo molecular evidence; no author-proposed nodes appear in this image. Corresponds to Section 2. HPV, human papillomavirus; TAP-1, transporter associated with antigen processing 1; APM, antigen-processing machinery; MHC-I, major histocompatibility complex class I; cGAS, cyclic GMP-AMP synthase; STING, stimulator of interferon genes; IFN, interferon; TAM, tumor-associated macrophage; MDSC, myeloid-derived suppressor cell; Treg, regulatory T cell; LAG3, lymphocyte-activation gene 3; LN, lymph node; CRT, chemoradiotherapy.

Chronic infection with high-risk HPV sets the starting state of the cervical TIME. Years of viral persistence impose a form of immune editing on the tissue (12, 13). The E6 and E7 oncoproteins do more than drive proliferation. They also remodel epithelial immunity. E7 lowers cell-surface major histocompatibility complex (MHC) class I, partly by downregulating the antigen-processing machinery (APM), including TAP-1. This weakens CD8^+^ T-cell recognition, while leaving the cell more exposed to natural killer cells (14, 15). HPV proteins also disturb the wider immune-signaling and DNA-damage-response proteome of keratinocytes (16). They blunt innate nucleic-acid sensing as well. Cutaneous HPV E6, for example, impairs the cGAS–STING pathway. Similar interference is reported across papillomaviruses, including downregulation of the same interferon-inducible genes by high-risk α-types (17). The result is a tissue selected, before any therapy, for low antigen visibility and a muted type I interferon tone (**Figure 2**).

Against this viral backdrop, the composition of the baseline TIME still predicts outcome. Higher intratumoral densities of CD8^+^ T cells, and of certain CD14^+^ myeloid populations, are associated with longer survival in cervical carcinoma (18); a pre-existing effector infiltrate is thus prognostically favorable. Immune suppression, by contrast, is spatially organized and extends beyond the primary tumor. Nodal metastases arise within delineated fields of immune suppression in the pelvic lymph-node catchment (19), so the baseline immune state is better read as a regional property than as a feature of the tumor core alone. Much of the suppressive machinery is already active at diagnosis: regulatory T cells (Tregs) and immunosuppressive myeloid cells populate the untreated tumor (20), and inhibitory receptors such as LAG3 are expressed on intratumoral Treg and CD8^+^ T cells before any therapy (21). Checkpoint ligand expression differs by histology, with distinct PD-L1 and stromal patterns in HPV-independent adenocarcinoma (22), while other regulators such as CMTM6 influence T-cell recruitment and tumor-cell behavior in glandular disease (23). Cellular senescence and senescence-associated secretory phenotype (SASP) programs, common to many tumor microenvironments, further shape this pre-treatment state (24).

This has two implications. Baseline PD-L1 combined positive score (CPS) and single-lineage densities are useful but only partial predictors, because they describe the initial conditions of a system that CRT then perturbs. HPV has already lowered antigen presentation and dampened interferon tone, so a tumor’s capacity to be “woken up” by CRT, meaning its ability to restore MHC expression and interferon signaling, may matter as much as its starting infiltrate. Viral burden adds a quantitative axis to this picture. A high HPV viral load independently predicts poor prognosis and marks a more immunosuppressive, exhaustion-prone microenvironment (25). Antigen quantity and immune quality are therefore coupled at baseline rather than independent, and the tumors that carry the most viral antigen are not necessarily the ones best placed to respond to it. That capacity is the subject of the next section.

### 2.1 Before chemoradiotherapy: induction (neoadjuvant) checkpoint blockade as a distinct timing strategy

A third timing option sits between the baseline state just described and the priming window that follows, and a review organized around timing would be incomplete without it. Induction dosing (also termed neoadjuvant or window-of-opportunity dosing) delivers blockade before chemoradiation begins, instead of concurrently with CRT or as post-CRT maintenance. The theoretical case rests on two arguments already developed above. The first is that the compartment on which checkpoint blockade acts is still intact. Radiation-induced lymphopenia has not occurred, the pelvic nodal basins that serve as priming sites have not been irradiated, and cisplatin has not yet been given. An induction dose therefore meets the largest and most functionally diverse effector pool the patient will have at any point in the treatment course (Section 4). The second comes from preclinical models of resectable disease. There, neoadjuvant checkpoint blockade generated a larger and more durable tumor-specific CD8+ T-cell response, and better eradication of distant micrometastatic disease, than the same agents given after removal of the primary tumor; the magnitude of the peripheral tumor-specific response predicted outcome (26). Transposed to chemoradiation, this logic predicts that induction ICI would pre-activate the tumor immune microenvironment. Radiation-induced antigen release would then meet an already-expanded and already-de-repressed T-cell population, instead of having to generate one inside a compartment that pelvic irradiation is dismantling at the same time.

Direct human evidence in cervical cancer now exists. It supports the mechanistic premise and leaves the clinical question open. In the GINECO COLIBRI window-of-opportunity phase II trial, 40 women with locally advanced cervical carcinoma received one cycle of nivolumab plus ipilimumab before standard chemoradiation, followed by nivolumab maintenance. The primary endpoint was met. Multiplex immunofluorescence on paired biopsies showed a significant increase in the intratumoral CD8+/FOXP3+ ratio and in proliferating CD8+ T-cell density after induction alone, with a significant rise in a 27-gene signature of immunologically active tumors (27). The trial therefore shows, in cervical tissue and before any radiation, that checkpoint blockade can shift the TIME toward an activated state. It cannot show that induction is the preferable schedule. It was single-arm, it did not randomize induction against concurrent dosing, and its efficacy signal is inseparable from the subsequent chemoradiation and maintenance. Three qualifications follow. Induction and concurrent dosing are not alternatives but are potentially complementary: on the priming-window logic of Section 3, an induction dose enlarges the effector pool that concurrent dosing then protects during irradiation, so the plausible optimum may be induction plus concurrent rather than either alone. Delaying definitive chemoradiation to fit an induction phase carries an oncological cost in a disease where time to treatment completion is prognostic, and that cost bounds how long such a phase can be. The safety of front-loading dual checkpoint blockade before pelvic irradiation is also not established at scale (Section 7.1). Adding PD-1 blockade to chemoradiation already raises potentially immune-mediated adverse events from 17% to 39% (4). A CTLA-4-containing induction phase adds a further burden and adds it before irradiation begins rather than alongside it. An event arising in that window may postpone chemoradiation, and treating it with corticosteroids would, on the priming logic above, blunt the effector expansion that induction was given to produce. Three design consequences follow. An induction phase should be capped at a single cycle, as in COLIBRI (27), with the start of chemoradiation fixed by protocol and not left to discretion. Feasibility should be co-primary with any immunological endpoint, not a secondary observation. The natural metric is the proportion of patients completing radiotherapy within the standard 56-day window, used in the randomized schedule comparison of Section 7. Single-agent PD-1 blockade may also be the better induction agent when definitive pelvic irradiation follows, because the incremental priming from CTLA-4 blockade has not been separated from its added toxicity in this setting. We therefore treat induction as a third timing arm that a factorial trial of schedule should ideally include. Its absence from both pivotal trials is a further reason the concurrency-versus-maintenance question remains under-determined by existing data.

## 3 Early chemoradiotherapy: the immunogenic priming window

The opening days to weeks of CRT constitute a window in which cytotoxic therapy can convert an immunologically quiet tumor toward an immunogenic one. The mechanistic core is the sensing of therapy-induced DNA damage. Ionizing radiation generates cytosolic double-stranded DNA that activates the cGAS–STING pathway and drives a type I interferon (IFN) response required for radiation-induced priming of CD8^+^ T-cell immunity in preclinical models (28); indeed, the antitumor efficacy of radiotherapy itself depends on host type I interferon signaling and the enhanced cross-priming it confers on tumor-infiltrating dendritic cells (29). The magnitude of this response is tuned by DNA-handling enzymes: the exonuclease TREX1, induced at higher fraction sizes, degrades cytosolic DNA and limits interferon output, providing a mechanistic rationale for fractionation choices in radioimmunotherapy (30). Parallel death-and-sensing programs, including necroptotic ZBP1–MLKL signaling and pharmacologic engagement of the DNA-damage response (for example ATR inhibition), can amplify intratumoral STING activation and immunogenicity (31, 32). In cervical cancer models specifically, cGAS–STING signaling is attenuated yet remains druggable: pharmacologic STING activation raises type I interferon output, expands tumor-infiltrating CD8^+^ T cells and dendritic cells, and restrains tumor growth (33), direct in-model evidence that the priming axis central to this section is intact in the disease itself.

Radiation also increases the antigenic visibility of tumor cells. It upregulates MHC class I and adhesion/costimulatory molecules, an effect documented in cervical cancer cells decades ago (34) and mechanistically linked to therapeutic response in more recent squamous-carcinoma models (35), and it can trigger immunogenic cell death with release of tumor antigens and danger signals that license dendritic-cell priming (36, 37). At the systemic level, these processes underlie the abscopal phenomenon, in which localized irradiation combined with immune activation can generate responses at distant sites; although clinically infrequent, the abscopal literature provides proof of principle that radiation can convert a tumor into an *in situ* vaccine (38, 39).

The chemotherapy partner is not immunologically neutral. Cisplatin has its own immunomodulatory effects. It enhances antigen presentation, sensitizes tumor cells to cytotoxic lymphocytes, and shifts the myeloid and effector compartments in ways that may complement radiation-induced priming (40, 41). Together, radiotherapy plus platinum chemotherapy supplies much of what a virally silenced tumor lacks. In principle it restores MHC expression and renews interferon signaling, while dying cells release tumor antigen (34, 35, 40, 41). This fits the general rationale for pairing radiation with immune activation (42).

Two caveats temper this picture. Much of the mechanism comes from preclinical and non-cervical models. Provenance determines how much weight each mechanistic claim can bear. We therefore set out explicitly which links in this chain have been validated in human cervical material, and which are imported from other systems. Validated in cervical cells, tissue or patients: HPV E7-mediated loss of surface MHC class I and of antigen-processing machinery including TAP-1 in cervical carcinoma (14, 15); HPV E6/E7 perturbation of the immune-signaling and DNA-damage-response proteome of human keratinocytes, together with E6-mediated impairment of cGAS–STING and downregulation of interferon-inducible genes by high-risk alpha-types (16, 17); radiation-induced upregulation of MHC class I and adhesion/costimulatory molecules on cervical carcinoma cells (34); attenuated but pharmacologically restorable cGAS–STING signaling in cervical cancer models, where STING agonism raises type I interferon output and expands tumor-infiltrating CD8+ T cells and dendritic cells (33); and, at the level of on-treatment outcome, intratumoral immune activation, loss of local immune response in resistant tumors, and HPV E6/E7-responsive clonal expansion in patients undergoing cervical CRT (43–46). Extrapolated from non-cervical systems, and therefore graded as inferential here: the requirement for cGAS–STING and host type I interferon in radiation-induced CD8+ priming (28, 29); TREX1-dependent, fraction-size-dependent tuning of interferon output (30); ZBP1–MLKL necroptotic signaling and ATR-inhibitor amplification of STING activation (31, 32); MHC class I upregulation as a mechanism of therapeutic response (35); immunogenic cell death with calreticulin exposure and HMGB1–TLR4 signaling (36, 37); the abscopal phenomenon (38, 39); and the immunomodulatory contribution of cisplatin (40, 41). The distinction is carried through the rest of this review. Every Stage-2 biomarker candidate in Section 8 that derives from the extrapolated column is graded C rather than A in **Table 3**. Direct evidence that CRT restores antigen presentation and interferon tone in cervical patients over time is still missing. Closing that translational gap is what the longitudinal human studies in Section 4 begin to do. There is a second limit. The same DNA-sensing machinery that primes immunity is subject to negative feedback and can be subverted from within the cell. Early priming therefore does not guarantee sustained activation. That tension defines the mid-treatment window.

## 4 Mid-treatment: activation meets attrition

**Figure 3:**
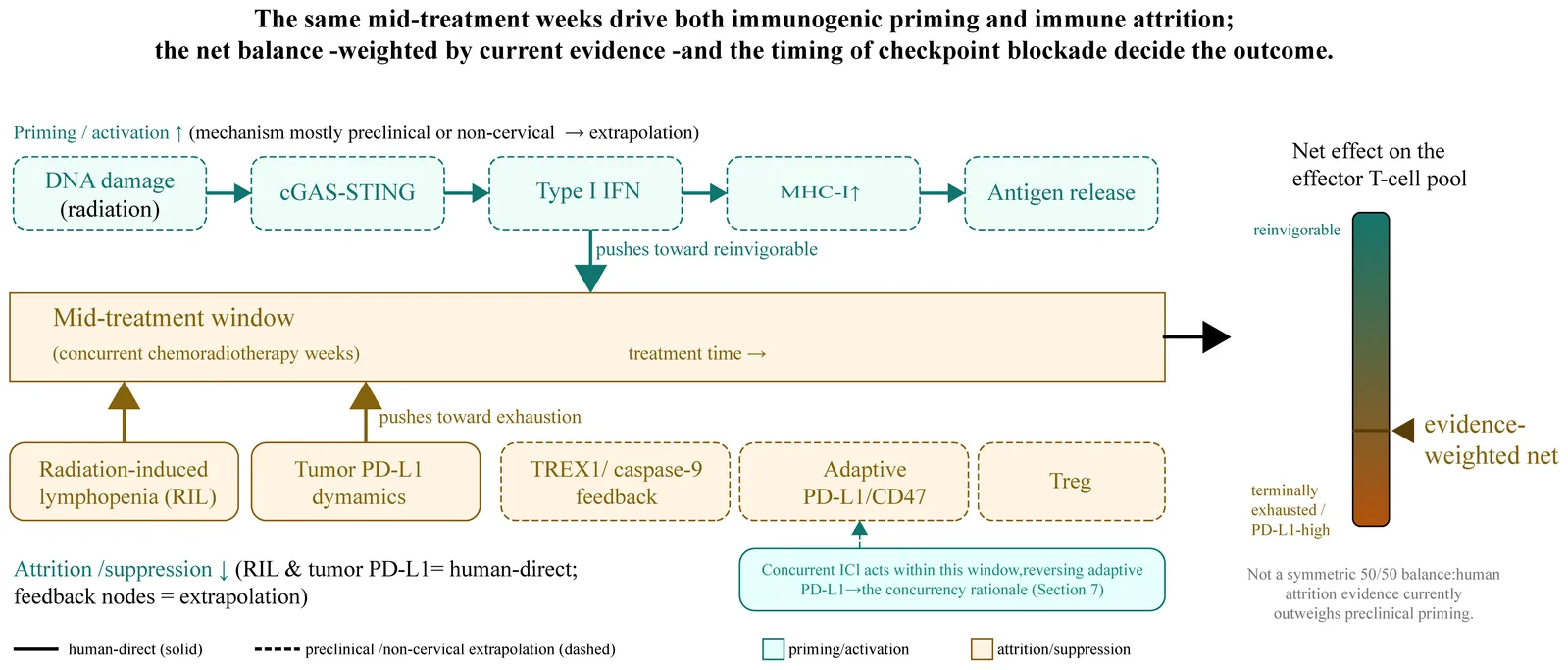
Mid-treatment tension: the same weeks drive both immunogenic priming and immune attrition. A central mid-treatment time band separates an upper priming process chain (DNA damage → cGAS–STING → type I interferon → MHC-I upregulation and antigen release) from a lower attrition chain (radiation-induced lymphopenia and tumor PD-L1 dynamics, plus TREX1/caspase-9 feedback, adaptive PD-L1/CD47, and Treg enrichment); both converge at right into a net-effect readout spanning reinvigorable to terminally exhausted/PD-L1-high. Opposed force arrows indicate that priming pushes the net toward reinvigorable while attrition pushes it toward exhaustion, and a callout marks where concurrent checkpoint blockade would act within this window to reverse adaptive PD-L1 (the concurrency hypothesis, Section 7). The blockade shown there is a predicted rather than a demonstrated intervention point. Evidence status is encoded throughout: radiation-induced lymphopenia and tumor PD-L1 dynamics are human-direct (solid), whereas the priming nodes and the intrinsic-feedback nodes derive largely from preclinical or non-cervical models (dashed) and are shown as mechanistic hypotheses under test by the human longitudinal data of **Table 1**, not as established cervical events. The composition is deliberately asymmetric rather than a symmetric 50/50 balance: because human evidence for attrition currently outweighs the largely preclinical priming mechanisms, the net readout tilts toward exhaustion until longitudinal human priming data mature. Color semantics: teal, priming/activation; amber, attrition/suppression. Corresponds to Sections 3–4. Abbreviations: CRT, chemoradiotherapy; ICI, immune checkpoint inhibitor; cGAS–STING, cyclic GMP-AMP synthase–stimulator of interferon genes; IFN, interferon; MHC-I, major histocompatibility complex class I; RIL, radiation-induced lymphopenia; PD-L1, programmed death-ligand 1; TREX1, three-prime repair exonuclease 1; CD47, cluster of differentiation 47; Treg, regulatory T cell.

If any single phase decides whether CRT–ICI succeeds, it is the middle of treatment, when opposing forces act at once. This window holds the clearest longitudinal human data. That clarity is only relative. The direct human evidence rests on a handful of small paired-sampling studies, each covering tens to about a hundred patients (**Table 1**). The trajectory reconstructed below is therefore best read as a working hypothesis built on limited but high-value data, not as an established sequence (**Figure 3**).

Intratumoral activation. Serial sampling during chemoradiation for cervical cancer demonstrates active immune engagement while treatment is ongoing. One prospective study of intratumoral kinetics found measurable activation of tumor-infiltrating immune cells over the treatment course (43). A prospective clinical trial that tracked both compartments documented dynamic changes in the local and systemic TIME during concurrent CRT. These included shifts in effector and regulatory populations that baseline sampling alone would have missed (44). These data establish, in patients, that the tumor is being immunologically remodeled while therapy is delivered.

Intratumoral attrition and resistance. Activation, however, is opposed by several convergent programs. Fractionated radiotherapy drives adaptive upregulation of PD-L1 in the irradiated tumor, and this acquired resistance can be overcome by concurrent PD-L1 blockade in preclinical models (extrapolated; level C), a finding that provides the single most direct rationale for scheduling ICI concurrently rather than after radiation (47). Tumor cells actively suppress radiation-induced immunity through cell-intrinsic circuits such as caspase-9 signaling, which restrains the very DNA-sensing response that radiation triggers (48). Regulatory T cells are a key brake on radiotherapeutic anti-tumor responses, and their persistence relative to effector cells can tilt the local balance toward suppression (49), and inhibitory receptors beyond PD-1 (for example on exhausted CD8^+^ T cells) accumulate as the effector response matures (50). The critical clinical correlate in cervical cancer is provided by paired pre-/on-treatment tumor analysis: tumors destined for treatment resistance showed a decreased local immune response during chemoradiation together with retained HPV oncogene expression, directly linking a failure of on-treatment immune activation to poor outcome (45).

Peripheral attrition: radiation-induced lymphopenia. The second arm of attrition is systemic. Pelvic radiotherapy delivers substantial dose to circulating lymphocytes and to pelvic marrow, producing radiation-induced lymphopenia (RIL) that is common, often severe, and prognostically adverse. Severe RIL is associated with worse overall survival across solid tumors (51), is specifically linked to inferior survival in cervical cancer (52) and has been argued to act as a causal mediator of poorer outcomes after chemoradiation, not merely a marker (53). Systemic inflammatory indices also shift during definitive CRT in cervical cancer, tracking tumor burden and treatment (54). This matters mechanistically for immunotherapy. When the effector pool is depleted and does not recover, the T cells that ICI is meant to reactivate are absent, and blockade has nothing to act on. In lung-cancer models, a bifunctional anti-PD-L1/TGF-β agent retained efficacy even when lymphocyte recovery was disrupted (55). The window thus does two contradictory things at once. It recruits and activates T cells inside the tumor while draining them from the circulation. This is a defining and still underappreciated feature of CRT, and a recognized obstacle to combination radio-immunotherapy (56, 57). Longitudinal profiling in cervical CRT shows that the depletion is lineage-selective. It reduces CD4^+^ T-helper and B cells most, while relatively sparing CD8^+^ T and natural killer cells (58). In the PET-guided INTERTECC trial it significantly reduced acute high-grade hematologic toxicity, though this did not translate into a progression-free survival benefit (59). RIL is the most modifiable obstacle in this window, so the mitigation strategies are set out here in full. Three are in development, at different stages of maturity. Bone marrow-sparing planning is the most developed. Guided either by functional imaging of active marrow or by anatomical atlas, it reliably lowers pelvic marrow dose and acute hematologic toxicity in cervical cancer and is deliverable with volumetric-modulated arc therapy (VMAT) on standard platforms (59). It has also prompted the argument that pelvic marrow should be treated as a formal organ at risk in pelvic nodal radiotherapy (60). Its ceiling is that marrow dose is only one determinant of the lymphocyte nadir. A dynamic lymphocyte-flow model built for locally advanced cervical cancer predicts that adding a 3 Gy marrow-sparing objective to a VMAT plan barely shifts the nadir, because most lymphocyte kill occurs in blood transiting the irradiated pelvis rather than in marrow reserve (61). Reducing the integral dose delivered to circulating blood addresses that dominant term directly and is predicted to have a much larger effect. In the same cervical model, intensity-modulated proton therapy raised the relative lymphocyte nadir from roughly 33% to 48% of baseline in the blood compartment, and from 48% to 63% in total body, compared with VMAT (61). The third strategy alters the temporal and geometric structure of exposure: fewer fractions, shorter overall treatment time, smaller elective nodal volumes, and planning objectives written on the circulating blood pool rather than on marrow. Each reduces the number of times the whole blood volume is irradiated. None of the three has yet been shown to improve immunological or oncological outcome, and the proton and fractionation data remain dosimetric or modeled rather than clinical. Lymphocyte sparing has never been tested where the present framework predicts it should matter most, namely as a co-intervention within CRT–ICI. If the effector pool is the substrate on which checkpoint blockade acts, then a blood- or marrow-sparing technique is not supportive care but a candidate efficacy modifier. The informative experiment is a CRT–ICI trial that randomizes or stratifies by lymphocyte-sparing technique, while measuring the nadir as a prespecified mediator. Sparing lymphoid tissue is necessary, but it may not be sufficient for an immunologic gain.

The exhaustion and clonality trajectory. Cell counts capture only part of the story. The quality and clonal structure of the surviving T-cell response also change across the window, in ways baseline sampling cannot capture. Serial tracking of the peripheral T-cell receptor (TCR) repertoire during concurrent CRT in cervical cancer shows a measurable fall in clonal diversity over the treatment course (44). This fall in diversity most likely reflects circulating clones being lost to lymphodepletion faster than newly recruited, antigen-experienced effectors are exported. The clones that matter here can be tracked through their antigen specificity. Serial deep sequencing of the intratumoral repertoire during chemoradiation with brachytherapy showed expansion of HPV E6/E7-responsive clones over the treatment course in most patients (46), and the patients whose repertoire failed to remodel were those who later recurred. In humans, then, the on-treatment window is a period of active, antigen-specific clonal selection whose success or failure carries prognostic weight. The inhibitory-receptor program widens over the same interval, extending beyond PD-1. LAG3 is expressed on intratumoral regulatory and CD8^+^ T cells in cervical cancer (21), and terminally exhausted CD8^+^ populations accumulate after fractionated radiation in preclinical models (50). Because an interferon-rich milieu can itself drive compensatory inhibitory-ligand expression, a tumor that looks transcriptionally responsive may be arming several brakes at the same time. The therapeutic implication is clear. Single-axis blockade with anti-PD-1 or anti-PD-L1 may engage only one of several redundant checkpoints once the microenvironment has begun to diversify its inhibitory repertoire, which argues both for scheduling blockade early, before the compensatory program is in place, and for combination checkpoint strategies in some tumors. The mid-treatment window therefore fixes the size of the effector pool and provisionally sets its functional fate, whether it remains reinvigorable or becomes terminally exhausted.

An open question: target availability and drug disposition under profound lymphodepletion. The account above, and the literature it rests on, treats RIL as a problem of effector-cell number, with too few T cells for blockade to reinvigorate. A second possibility is largely unexamined, and it is pharmacological rather than cellular. Anti-PD-1 and anti-PD-L1 antibodies are given at fixed or weight-based doses calibrated in populations with intact lymphoid compartments, and their disposition is target-mediated. In humanized-mouse PET imaging, 89Zr-labeled pembrolizumab accumulated mainly in PD-1-expressing lymphoid tissue (spleen, lymph nodes and bone marrow), with only modest tumor uptake. That lymphoid sink could be saturated with unlabeled antibody without changing tumor uptake (62). Profound, treatment-induced depletion of those same compartments would be expected to shrink the peripheral antigen sink, and so raise free drug exposure, while reducing the number of intratumoral targets the drug is meant to engage. Whether the net effect on intratumoral receptor occupancy is favorable, neutral or adverse is unknown. A related uncertainty concerns delivery rather than dose. Antibody penetration into solid tumors is limited by the binding-site barrier, whereby high-affinity binding to the first antigen encountered after extravasation confines drug to perivascular regions at subsaturating doses (63). An irradiated cervical tumor is a poorly characterized setting for that process, with treatment-altered vascular permeability and interstitial pressure, and with a PD-L1 expression pattern that radiation itself upregulates (47). If fixed-dose blockade achieves inadequate occupancy at the tumor bed in exactly those patients whose lymphoid compartments are most depleted, then the failure mode attributed above to an absence of cells to act upon would be compounded by an absence of drug where the cells are. Distinguishing the two is technically feasible: receptor-occupancy assays on the T cells that remain, immuno-PET with labeled checkpoint antibodies, and on-treatment tumor pharmacodynamics. None of these has been deployed in cervical CRT–ICI. Until they are, dose and schedule in this setting rest on extrapolation from populations that were not lymphodepleted. We list intratumoral target occupancy in **Table 3** as a conceptual Stage-2 readout on that basis.

The same weeks that offer the best opening for immune engagement also impose the strongest opposing pressures, and patients diverge across them. Some emerge with an amplified, checkpoint-poised effector response; others emerge with an exhausted, lymphodepleted, PD-L1-high tumor. Which outcome prevails is, we argue, a major determinant of CRT–ICI benefit, and it cannot be read from baseline sampling alone.

One caveat deserves emphasis, because it recurs whenever these dynamics are turned into clinical decisions. The tumor-local and peripheral compartments are coupled, yet they are not equivalent. Within a single patient and time window, intratumoral activation and peripheral lymphodepletion can move in opposite directions, and several on-treatment intratumoral changes are not mirrored in the blood (43). The local and systemic compartments follow distinct trajectories during concurrent CRT (44). Peripheral readouts such as circulating lymphocyte counts, blood T-cell phenotypes, and ctHPV-DNA are attractive precisely because they can be sampled repeatedly, where tumor biopsies cannot. They remain, however, approximate surrogates for the local process rather than direct measures of it. How faithfully a blood signal reports the state of the irradiated tumor bed is itself an open, testable question. We return to it as a validation priority for the biomarker framework (Section 8) and future longitudinal designs (Section 10).

## 5 After treatment: clearance, persistent antigen, and adaptive resistance

The immune state established by the end of CRT plausibly governs whether residual disease is cleared or escapes. Two features distinguish cervical cancer here: a defined viral antigen whose persistence can be tracked, and adaptive resistance programs that mature as therapy withdraws its pro-inflammatory stimulus.

Viral antigen persistence and minimal residual disease. Because HPV DNA is tumor-specific, ctHPV-DNA offers a quantitative, blood-based readout of residual viral-antigen-bearing disease. Serial ctHPV-DNA during and after chemoradiation for LACC tracks treatment response, and persistence or failure to clear is associated with recurrence risk (64, 65). Technical maturation has improved sensitivity and specificity for this analyte, through droplet digital and nanoplate digital PCR assays (validated in HPV-associated cancers) and HPV-integration-site markers (66–68), and dynamic monitoring during concurrent CRT is now being evaluated as a real-time efficacy readout (69), within a growing body of work on circulating cell-free DNA in cervical cancer (70). In the phase III CALLA cohort, ultrasensitive ctDNA detection and tracking predicted relapse and survival, establishing molecular residual disease (MRD) as a prognostic axis even where the therapeutic intervention itself was negative (71). The complementary tissue observation is that retained HPV E6/E7 expression in the tumor during therapy marks resistance (45), linking the circulating and tissue views of antigen persistence.

Adaptive resistance. As CRT ends, the interferon-rich, immunogenic milieu of the priming window gives way to counter-regulation. Adaptive resistance circuits engaged during radiation can dominate once therapy-induced sensing subsides; one such circuit is the ATR-driven upregulation of CD47 and PD-L1 that restrains radiation-induced immune priming and abscopal responses, shown preclinically in colorectal models (72) and therefore extrapolated (level C). In cervical models, restoring innate activation (for example with STING agonists) can re-inflame the TIME and overcome anti-PD-1 resistance, indicating that post-treatment resistance is, at least partly, a reversible deficit of innate stimulation rather than a fixed state (73). Distinct tumor-cell states can also actively enforce post-treatment immune escape: chemoradiotherapy-induced ACKR2^+^ tumor cells drive CD8^+^ T-cell senescence and cervical cancer recurrence, providing a concrete cellular mechanism by which the tumor converts a treated bed into an immune-privileged niche (74). Studies of the immune microenvironment in the recurrent and immunotherapy-exposed setting further show how the TIME is reshaped by prior treatment, with implications for salvage strategies (75).

The post-treatment state is thus best read as a competition between immune surveillance of viral-antigen-bearing residual cells and the tumor’s adaptive-resistance and antigen-persistence programs. This is precisely the window that maintenance ICI, as delivered in KEYNOTE-A18, is positioned to influence, and it is where ctHPV-DNA-guided, adaptive immunotherapy strategies are most plausible.

The competition also has a temporal asymmetry that bears on how maintenance should be deployed. The immunogenic stimulus of CRT is transient: type I interferon tone, MHC-I re-expression, and antigen release peak during treatment and then subside, whereas the tumor’s counter-regulatory programs (adaptive PD-L1/CD47 induction, ACKR2^+^-driven CD8^+^ senescence, and the reconstitution of a suppressive myeloid and regulatory compartment) mature precisely as the pro-inflammatory drive withdraws (72, 74). Maintenance checkpoint blockade should therefore act on a microenvironment that is drifting toward suppression. This frames why its benefit may depend on the state a patient carries out of the mid-treatment window. A tumor that has generated and exported an antigen-specific effector pool offers maintenance ICI a population to protect, whereas one that failed to prime, or that emerged lymphodepleted, offers the antibody little to release. On this reading, maintenance is not a uniform intervention but a state-dependent one, and the same molecular-residual-disease readouts that detect persistence could in principle triage it. Sustained ctHPV-DNA clearance identifies patients in whom surveillance is winning and de-escalation may be safe. Persistence or rising ctHPV-DNA instead marks the adaptive-resistance state, in which maintenance monotherapy is least likely to suffice and escalation becomes the rational hypothesis to test, whether combination checkpoint blockade or the antigen-directed strategies discussed in Section 9. That ctHPV-DNA kinetics can, in cervical cancer specifically, separate these trajectories is the strongest available argument for building post-treatment biomarker triage into future maintenance trials rather than treating all patients uniformly.

### 5.1 HPV-independent histologies: the boundary of an antigen-anchored model

The framework developed to this point is anchored on a viral antigen. That anchoring is what makes cervical cancer unusually easy to track: a tumor-specific antigen (E6/E7), a blood-based residual-disease analyte derived from it (ctHPV-DNA), and an effector population definable by its specificity. It is also the framework’s boundary. Under the 2020 World Health Organization classification, endocervical adenocarcinomas are divided into HPV-associated and HPV-independent categories. The latter comprises principally gastric-type adenocarcinoma, together with clear-cell, mesonephric and endometrioid variants. HPV-independent tumors are not a negligible fraction of cervical adenocarcinoma. In locally advanced disease they carry significantly worse progression-free and overall survival than their HPV-associated counterparts (76). Gastric-type adenocarcinoma in particular is aggressive, frequently p53-aberrant with variable p16 expression, and recurs in roughly a quarter to a third of patients (77). For this group, three components of the temporal model fail, each in a different way.

First, the antigen-anchored readouts do not transfer. ctHPV-DNA is undefined where there is no viral genome, so Stage 3 of the framework would have to be re-derived on tumor-informed, mutation-based ctDNA. That is feasible in principle. It is indirectly supported by the CALLA correlative analysis, in which the tumor-informed assay rather than its HPV-specific component carried most of the prognostic signal (71). It would nonetheless require individualized panel design, and prospective validation in a rarer and more heterogeneous population. Expansion of E6/E7-responsive clones (46) likewise cannot serve as the on-treatment specificity readout. Neoantigen-directed T-cell-receptor tracking is the analogue, and it is considerably less mature. Second, the baseline state differs in kind rather than in degree, and at least one marker inverts. Gastric-type adenocarcinoma shows sparse T-cell infiltration with abundant, predominantly M2-polarized macrophages in advanced stage. Its tertiary cells and structures (TLS) are immunosuppressive in composition, enriched for B cells, PD-1-negative CD8+ T cells and regulatory T cells, and associated with worse rather than better prognosis (78). This is the opposite of the direction reported for TLS in HPV-associated cervical cancer (79, 80), and it shows concretely that a marker’s directionality cannot be assumed to carry across histologies. Third, the priming premise itself is weaker. The case for CRT as an *in situ* vaccine in Section 3 rests substantially on converting a virally silenced but antigen-rich tumor into a visible one. HPV-independent tumors have no such defined antigen, an unclear mutational antigen load, and are clinically less responsive to definitive chemoradiation. One series reported more favorable progression-free survival for a surgery-based than a definitive-chemoradiation approach in HPV-independent locally advanced endocervical adenocarcinoma (76). We therefore restrict the substantive claims of this review to HPV-associated cervical carcinoma, the population enrolled in both pivotal trials. HPV-independent disease is a separate problem. It requires its own temporal model, its own residual-disease analyte and, given the prognostic weight of an immunosuppressive TLS phenotype, potentially an inverted reading of several baseline markers. The first step is descriptive rather than a trial: a longitudinal cohort with paired pre-, on- and post-CRT sampling in HPV-independent adenocarcinoma, which at present does not exist.

## 6 Spatial and single-cell evidence

High-resolution technologies convert the time axis from an inference into an observable and test whether the states above have distinct cellular architectures. Single-cell dissection of cervical cancer has defined the key immune subsets of the TIME and their functional programs (81), and multi-omic analyses have resolved recurrent cellular ecosystems with biological and clinical relevance, including immune-inflamed and immune-excluded configurations (82), with immunosuppressive SPP1^+^ myeloid niches marking progression (83). Integrated single-cell and spatial studies link HPV-associated features to prognostic signatures (84), and single-cell transcriptomics has resolved the prognostic contribution of specific populations, including HPV16-positive macrophages (85).

Spatial organization, not just composition, discriminates outcomes. Immunosuppressive niches formed by cancer-associated fibroblasts (CAFs) and myeloid cells recur across the TIME. In cervical cancer, a CD54+ inflammatory-CAF/ITGAL+-macrophage niche drives immunosuppression via a CXCL8–PD-L1 axis (86), and IDO-expressing LAMP3+ dendritic cells synergize with regulatory and exhausted T cells to remodel the suppressive microenvironment (87). Work across tumor types extends the point, though only by extrapolation (level C). It is the spatial architecture of myeloid and T cells, rather than their bulk density, that governs immune evasion and clinical outcome (88). Nodal remodeling by fibroblast-myeloid niches that support metastatic tolerance carries the same principle into the regional compartment (89). This echoes the fields of immune suppression seen histologically at baseline (19).

Tertiary lymphoid structures add a further layer, both prognostically important and time-sensitive. In cervical cancer, the distribution and maturity of TLS predict recurrence-free survival. They generally associate with stronger anti-tumor immunity and a favorable prognosis (79, 80), in line with the wider gynecologic literature (90). TLS are context-dependent, however. Fatal cases show high densities of Tregs and polymorphonuclear myeloid-derived suppressor cells (PMN-MDSCs) within the TLS (91). The same structure can therefore be protective or permissive, depending on what it contains. For this review, the key development is that spatial evidence is beginning to be read dynamically: spatiotemporal immune determinants of response to immune rechallenge have been described in advanced cervical cancer, connecting spatial architecture to the sequencing of immunotherapy lines (10). Together, single-cell and spatial data are consistent with the central hypothesis that the cervical TIME occupies discrete, remodelable states whose configuration, and trajectory, may govern response. On the same scale, only the serial spatial profiling of immune rechallenge (10) is longitudinal in cervical patients (level A). The cervical single-cell, niche and TLS analyses are cross-sectional (level B), and the cross-tumor architectural principle is extrapolated (level C).

## 7 Reconciling trial heterogeneity through time

The temporal framework offers a disciplined way to interpret why PD-(L)1 blockade added to CRT succeeded in KEYNOTE-A18 (4, 5) but not in CALLA (6) (**Table 2**), without collapsing the explanation onto a single variable. The two regimens differ in more than their target; they also differ in how exposure was structured. KEYNOTE-A18 used two protocol phases: five cycles of pembrolizumab 200 mg every three weeks during chemoradiotherapy, then fifteen cycles of 400 mg every six weeks as maintenance (4, 5). CALLA used one schedule throughout, durvalumab 1500 mg every four weeks with and after chemoradiotherapy for up to twenty-four cycles (6). Nothing in that regimen marked the end of irradiation. On the schedules alone, the three-week interval fits roughly one extra dose into the weeks of pelvic irradiation. The larger difference is structural. The pembrolizumab protocol tied a defined block of exposure to the chemoradiotherapy phase; the durvalumab schedule simply ran on across it. The results then differed in degree, not in direction: progression-free survival hazard ratios were 0.70 (95% CI 0.55–0.89, p=0.0020) and 0.84 (95% CI 0.65–1.08, p=0.17). Two further differences bear on population comparability rather than on schedule: the International Federation of Gynecology and Obstetrics (FIGO) staging edition used (2009 in CALLA, 2014 in KEYNOTE-A18) and the permitted platinum. That the negative trial’s estimate pointed the same way argues against a categorical difference in drug activity, and for placing timing among the correlated variables below, not ahead of them. The trials differed along at least seven axes that the preceding sections render biologically meaningful, and we present these as candidate contributors rather than established causes. The epistemic status of what follows needs to be unambiguous. None of the seven has been shown to cause the divergence between these two trials, and none is testable within the existing data. The trials differ on all seven at once, and neither collected the serial immunological readouts that would discriminate among them. Each axis below is a speculative candidate, generated by the biology of Sections 2–6 and consistent with the trials’ published design differences. The list is best read as a specification of what a future factorial or correlative-rich trial would have to measure, not as an account of what happened. Where the text below uses verbs such as conditions, alters or determines, they denote a hypothesized relationship and not a demonstrated one.

1. Antibody target and mechanism. Anti-PD-1 (pembrolizumab) and anti-PD-L1 (durvalumab) are not interchangeable; downstream biology and the balance of PD-L1 versus PD-L2 engagement differ, and MHC- and interferon-dependent determinants can confer differential sensitivity to distinct checkpoint axes (demonstrated for CTLA-4 versus PD-1 blockade in melanoma and applied here only by extrapolation) (92). This is a plausible contributor but, on current data, cannot be isolated from schedule and population effects.
2. Schedule and the concurrency principle. The strongest mechanistic prior is that ICI should coincide with the radiation-induced priming and PD-L1-upregulation window (Sections 3–4) (47). Differences in how concurrent and maintenance dosing were structured therefore bear directly on whether the drug was present when the tumor was most vulnerable (93, 94). Systematic comparison of sequential versus concurrent ICI–radiotherapy across tumors confirms that timing modifies benefit, though in that pan-tumor network meta-analysis it was sequential, not concurrent, dosing that was associated with better outcomes (95). Mechanistic modeling likewise predicts that optimal combinations depend on the timing of administration (96). It is the most explicit formal statement of the timing hypothesis available, and its conclusions are conditional in a way that matters here. Sakai and colleagues profiled serial tumor biopsies taken across a radiotherapy course in esophageal cancer, by single-cell RNA sequencing and spatial transcriptomics. They observed that the lymphocyte signal shifts from an innate to an adaptive program during treatment and quantified the accompanying rise in ligand-receptor interactions including PD-1–PD-L1, CTLA-4–CD80/86 and TIGIT–PVR. Fitting these dynamics, their mathematical model predicted the efficacy of five ICI classes given at four timepoints. It concluded that anti-PD-1/PD-L1 agents exert maximal effect when given concurrently with radiotherapy, whereas anti-CTLA-4 and anti-TIGIT agents are predicted to be equally or more effective when given concurrently or adjuvantly (96). Two assumptions bound that prediction and limit its transfer to the present setting. The interaction dynamics were derived in esophageal rather than cervical tumors. And the model represents efficacy as a function of checkpoint-ligand engagement, without explicitly representing radiation-induced lymphodepletion, which Section 4 identifies as the dominant competing process during pelvic irradiation. The model therefore supports the target-specific character of our prediction, namely that concurrency should matter for PD-(L)1 blockade in particular and less so for other checkpoint axes. It leaves open whether pelvic RIL is severe enough to override that effect in this disease.
3. Baseline immunity and PD-L1. Differences in enrolled risk profile and in baseline immune composition would, on this framework, be expected to alter the starting state and thus the room for on-treatment gain (Section 2) (7). CALLA speaks to this directly, and it is the strongest competitor to a timing account: progression-free survival favored durvalumab more clearly as tumoral PD-L1 rose. Among the 369 patients with a tumor area positivity score of at least 20%, the hazard ratio reached 0.62 (95% CI 0.42–0.91). On that basis the trialists concluded that the combination warrants further exploration in high-PD-L1 disease (6). The subgroup analysis was *post hoc* and cannot support a causal claim, but it does mean that population selection has to be weighed against schedule, not after it.
4. HPV antigen load and clearance. Viral antigen persistence versus clearance, now measurable via ctHPV-DNA (Section 5), defines the antigenic substrate available to reinvigorated T cells (45, 71).
5. Stage, nodal burden, and radiation field. Larger fields and higher nodal burden increase both tumor antigen mass and the volume of irradiated lymphoid tissue, coupling potential benefit to potential harm. This axis is the most purely speculative of the seven. No published analysis of either trial has related field size or nodal burden to an immunological endpoint, and the coupling is asserted here on dosimetric and biological grounds alone.
6. Radiation-induced lymphopenia. The severity of RIL plausibly conditions whether an effector pool survives to be unleashed by ICI (Section 4). RIL is consistently associated with worse survival after RT/CRT (51–53), although that evidence predates ICI combinations and the link to ICI benefit remains inferential; trials and platforms differ in pelvic/marrow dose and thus in expected lymphodepletion.
7. On-treatment immune trajectory. Ultimately, whether a given regimen elicited IFN/MHC-I-associated activation or myeloid/adaptive suppression during treatment would, if the framework is correct, be the integrating variable, and the one least captured by trial correlatives to date (43, 44). It has not been measured in either pivotal trial, so this axis is at present a conjecture and not a candidate with supporting data.

These seven axes, and not antibody class alone, are the dimensions on which the two pivotal trials demonstrably differ. The corollary is methodological: trials that collect serial tumor and blood samples, as emerging combination-CRT studies such as NICOL increasingly do (93), are required to convert these candidate explanations into mechanism. This reasoning also motivates active investigation of sequencing and of radioimmunotherapy in curative settings across tumor types (97–99).

A further trial that again differs on several axes at once cannot isolate any single one. The informative design holds the backbone and population fixed and varies a single variable, most tractably the timing of blockade relative to radiation, while collecting the on-treatment readouts (interferon/MHC-I activation, RIL depth, ctHPV-DNA kinetics) that report which arm of the priming-versus-attrition contest prevailed. At this point the concurrency principle yields a directional and testable prediction. Fractionated radiation induces PD-L1 during treatment, and concurrent blockade reverses that acquired resistance in models (47). A regimen that delivers effective blockade inside the priming window may therefore outperform one that delivers the same total exposure mostly as post-CRT maintenance. Three constraints bound this prediction, and they determine what it is and is not derived from. First, it is not an inference from KEYNOTE-A18 and CALLA. Both trials gave PD-(L)1 blockade concurrently with chemoradiation and continued it as maintenance. Neither contains a contrast that isolates window dosing from maintenance dosing, and no comparison between them licenses any conclusion about the superiority of either schedule component. The prediction derives instead from three other sources: the preclinical demonstration that fractionated radiation induces PD-L1 and that concurrent, not sequential, blockade reverses that acquired resistance (47); the ligand-dynamics modelling described above (96); and the induction data of Section 2.1 (27). All three are mechanistic or model-based. None is a randomized clinical comparison of schedule in cervical cancer. Second, the best available randomized-evidence synthesis points the other way. In a network meta-analysis of sequential versus concurrent ICI–radiotherapy across solid tumors, it was sequential administration that was associated with superior outcomes (95). That analysis is not restricted to cervical cancer, does not isolate pelvic irradiation, and aggregates heterogeneous schedules and disease sites. It is nonetheless randomized-trial-derived evidence in direct tension with our mechanistic prior, and it must be weighted accordingly: on the present evidence base, the concurrency hypothesis is the least well supported of the two positions. The one randomized comparison of concurrent versus sequential pembrolizumab within cervical chemoradiation was a phase II study powered for safety and feasibility rather than efficacy, and it found no difference in progression-free or overall survival between the two schedules (100). That result is early and underpowered, but its direction is null, which is a further caution against treating concurrency as established. Third, the prediction is explicitly conditional and does not apply outside its premise. It holds only for patients who mount demonstrable on-treatment immune activation without catastrophic lymphopenia, and it makes no claim for patients who fail to prime, or in whom pelvic RIL is severe. Where severe RIL is common, the average effect of concurrent dosing could be null or negative even if the prediction were correct within the conditioning subgroup. We therefore label this a mechanistically motivated hypothesis that current clinical evidence does not support and in one respect contradicts, and whose value lies in being falsifiable by a trial that does not yet exist.

Two design features follow concretely from this reasoning. First, the concurrency-versus-maintenance ambiguity is separable only by a factorial allocation that crosses the timing of blockade (concurrent versus sequential) with its duration (maintenance versus none). A trial that varies both together, as the pivotal trials did, structurally confounds them. A two-by-two design, even if underpowered for survival, would for the first time attribute benefit to a schedule component rather than to a regimen as a whole. Second, the temporal framework predicts that benefit is concentrated in the subgroup that primes without catastrophic lymphodepletion. The efficient test is therefore not an all-comers comparison but a biomarker-stratified or adaptive-enrichment design. Such a design measures the on-treatment trajectory and enriches, or at minimum prespecifies subgroup analysis, for patients whose interferon/MHC activation rises and whose lymphocyte nadir stays above a threshold. This converts the on-treatment readouts from descriptive correlates into allocation variables, and it makes the framework of Section 8 falsifiable at the trial level: if timing and priming status do not modify the effect of blockade, the central prediction of this review is wrong.

Seen this way, the KEYNOTE-A18/CALLA divergence is not a paradox to be resolved by crowning one target. It is the expected result of two regimens meeting a moving immune system at different points. Until such correlative-rich trials report, the responsible reading is that target, schedule, population, and lymphodepletion are jointly capable of accounting for the divergence, that this remains an untested conjecture rather than an explanation, and that no published dataset licenses attributing it to antibody class alone.

### 7.1 The temporal toxicity profile: does early checkpoint exposure amplify acute radiation injury?

A framework built on the timing of immune engagement has to consider timing on the harm side as well, and the discussion so far has addressed efficacy alone. The concern is specific and biologically motivated. Pelvic chemoradiation injures rectal and sigmoid mucosa during the same weeks in which concurrent checkpoint blockade lowers the threshold for T-cell-mediated inflammation of the gut. Concurrent exposure might therefore produce more, or more severe, gastrointestinal toxicity than the same drug delivered after the mucosa has begun to heal. What the pivotal trials permit us to say is limited. In KEYNOTE-A18, grade 3 or higher adverse events of any kind occurred in 413 of 528 patients (78%) receiving pembrolizumab plus chemoradiation, versus 371 of 530 (70%) receiving placebo plus chemoradiation; anemia and decreased white-cell and neutrophil counts were most common, and potentially immune-mediated adverse events occurred in 206 (39%) versus 90 (17%) (4). In CALLA, serious adverse events occurred in 106 of 385 (28%) with durvalumab versus 89 of 384 (23%) with placebo. The most frequent grade 3–4 events in both arms were hematological (anemia 20% versus 15%; decreased white blood cells 10% versus 13%), and there were five treatment-related deaths with durvalumab versus one with placebo (6). Both trials therefore show a modest absolute increase in high-grade toxicity, and a substantial relative excess of immune-mediated events, when ICI is added to an already toxic backbone. In neither trial was the increment judged to compromise chemoradiation delivery.

The comparison this question actually requires is grade 3 or higher gastrointestinal toxicity disaggregated by treatment phase, concurrent versus maintenance. The published reports do not permit it and constructing it from aggregate figures would be misleading. Neither trial publishes adverse-event tables stratified by whether an event arose during the concurrent chemoradiation phase or during maintenance, and neither reports diarrhea or colitis rates separately by phase. The aggregate tables collapse an approximately six-week interval of concurrent pelvic irradiation together with up to two years of maintenance exposure. Over those intervals the background rates of radiation proctitis and of immune-mediated colitis differ substantially, and in opposite directions. A higher total incidence in an arm with longer total exposure is therefore uninformative about phase-specific risk and cannot be used to infer it. What limited direct evidence exists is reassuring but underpowered. In the randomized phase II comparison of concurrent versus sequential pembrolizumab with pelvic chemoradiation, treatment-related grade 3 events occurred in 43% and grade 4 events in 23% of 94 evaluable patients, with no grade 5 events; 80% completed radiotherapy within 56 days, and the authors concluded that both schedules were safe and feasible (100). Two implications follow. Clinically, the absence of a demonstrated phase-specific gastrointestinal signal should not be read as evidence of its absence, and the possibility that early ICI exposure aggravates acute radiation enteritis remains open. Methodologically, phase-resolved and organ-specific adverse-event reporting should become a standard requirement for CRT–ICI trials, on the same rationale that motivates serial correlative sampling. Events would be time-stamped relative to the end of external-beam radiotherapy and analyzed as incidence per exposure-week rather than as crude proportions over unequal intervals. A framework predicting that efficacy varies with timing predicts that toxicity does too, and only phase-resolved reporting can test either half of that claim.

## 8 A temporal biomarker framework

The observations above can be organized into a practical, three-stage biomarker framework. We present this explicitly as an author-proposed conceptual framework, aligning candidate biomarkers to the treatment phase at which they are informative; most components require prospective validation in cervical cancer specifically, and we flag the strength of evidence accordingly. **Table 3** is the framework’s visual summary. It sets each stage against its candidate marker, the sample required, the evidence level and the intended clinical use. **Figure 1** places the same three stages on the treatment trajectory, so the framework can be read as a matrix or along the time axis.

Stage 1: Baseline predictive markers (patient selection). Before treatment, the goal is to estimate both the starting immune state and the tumor’s capacity to be primed. Candidates include PD-L1/CPS and single-lineage densities (7), baseline MHC/interferon competence (extrapolated from melanoma) (92), multiplexed spatial architecture and TLS presence/maturity (10, 79, 80), and baseline peripheral immune composition, which in other tumors predicts ICI efficacy and toxicity (101). In other immunogenic tumors, the spatial arrangement of immune cells governs immune evasion and outcome and predicts checkpoint-blockade response more robustly than bulk density (88, 102), and the same principle plausibly governs whether a cervical tumor’s baseline infiltrate is positioned to be mobilized by CRT. The framework starts from one premise: no single baseline marker suffices. A composite of infiltrate, antigen-presentation competence, and spatial organization captures “primability” better than any of these alone.

Stage 2: On-treatment early-response markers (adaptive decision-making). The framework’s distinctive claim is that the mid-treatment window is measurable and decision-relevant. Because repeat cervical biopsy during CRT is not routine clinical practice, this stage prioritizes non-invasive, serially accessible readouts (blood and imaging) while treating on-treatment tissue readouts as largely research-grade and reserved for correlative trial cohorts. In cervical cancer specifically, most candidate readouts are extrapolated from other tumors rather than validated cervical markers. They include on-treatment interferon-γ–associated transcriptional activation (103, 104), dynamic peripheral T-cell analysis (105), non-invasive radiomic monitoring of the inflamed microenvironment (106), immune-cell-dynamic signatures that also track immune-related toxicity during chemoradio-immunotherapy (107), and early ctDNA molecular response, plausibly transferable to ctHPV-DNA during CRT (108, 109). The depth of radiation-induced lymphopenia is the one early signal with direct cervical human support (51, 53). Four Stage-2 quantities are concrete enough to prespecify: the absolute lymphocyte-count nadir during chemoradiation; an interferon-γ-associated transcriptional score at about week 3; the change in ctHPV-DNA from baseline to week 3; and the share of the intratumoral repertoire held by E6/E7-responsive clones. Each needs a threshold fixed before analysis, not derived from it. A structural caveat carries over from Section 4: the informative on-treatment signals that are easiest to obtain are peripheral, yet the process they are meant to report is tumor-local. The framework therefore treats these markers as accessible surrogates whose local–peripheral correspondence must be validated per marker, not assumed, which makes the paired sampling of tumor and blood a prerequisite for qualifying any Stage-2 readout rather than an optional add-on.

Stage 3: Post-treatment residual-disease markers (surveillance and maintenance guidance). After CRT, the priority is detecting molecular residual disease and adaptive escape. ctHPV-DNA clearance versus persistence is the leading candidate, supported by response-monitoring and relapse-prediction data in cervical cancer (64, 65, 69). The phase III CALLA analysis validated tumor-informed ctDNA as molecular residual disease predicting relapse and survival (71), with the HPV-derived signal one component, predictive mainly post-treatment. This is consistent with the broader principle that on-/post-treatment ctDNA clearance associates with benefit from checkpoint blockade, albeit with limited specificity, so that failure to clear is the more informative signal (110). Persistent ctHPV-DNA, or a residual ACKR2^+^/senescence-associated escape signature (74), would nominate patients for intensified or ctDNA-guided maintenance immunotherapy.

Mapped onto the trajectory, the framework yields a testable clinical hypothesis: patients who begin with a primable baseline, mount interferon/MHC-I activation on treatment without catastrophic lymphodepletion, and clear ctHPV-DNA afterward would define the population most likely to benefit from concurrent-plus-maintenance PD-1 blockade, while failure at any stage nominates a distinct interventional target (priming augmentation, lymphocyte sparing, or salvage immunotherapy, respectively).

The candidates within this framework do not carry equal evidential weight, and the framework is only useful if that is made explicit (formalized as the evidence-level column of **Table 3**). At present, only one axis rests on cervical-specific, longitudinal data: post-treatment molecular residual disease, read as ctHPV-DNA/tumor-informed ctDNA clearance versus persistence (64, 65), corroborated by within-randomized-trial evidence from CALLA, where a tumor-informed ctDNA assay predicted relapse and survival (71) (its HPV-specific component being predictive mainly after treatment). It thus approaches the threshold for prospective clinical qualification. Baseline PD-L1/CPS is validated as a treatment-selection marker in the metastatic setting but not as a predictor of CRT–ICI benefit in LACC. The remaining Stage-2 readouts (on-treatment interferon/MHC-I activation, spatial primability scores, dynamic peripheral T-cell signatures) are, in cervical cancer specifically, still at the level of mechanistic plausibility extrapolated from other tumors, and the composite “primability” index is frankly conceptual. Presenting it with the grading turns it into a research agenda, in which each cell of the matrix specifies the study needed to promote a candidate from hypothesis to qualified biomarker.

A framework without a stated route to qualification is not falsifiable in practice, so we set out the validation pathway each stage would require. Stage 3 is closest to qualification and needs a confirmatory rather than an exploratory design: a prospective, multicenter observational cohort of patients receiving standard CRT–ICI, with ctHPV-DNA and tumor-informed ctDNA sampled at fixed timepoints (baseline, end of chemoradiation, and 3, 6 and 12 months). The assay platform, the positivity threshold and the analytical validation would be fixed before enrolment. Recurrence would be the primary endpoint, and results would be blinded to treating clinicians so that management decisions do not confound the association. A cohort in the region of 300–400 patients would be needed to estimate a hazard ratio of the magnitude reported in the CALLA correlative analysis with useful precision. Stage 2 requires a different instrument, because its markers must be shown to be decision-relevant and effect-modifying, and not merely prognostic. The appropriate vehicle is a correlative sub-study nested within a CRT–ICI trial. It would collect paired tumor and blood before treatment and at approximately week 3, report the tumor-blood correspondence of each candidate marker as a prespecified analysis rather than assuming it, and prespecify lymphocyte nadir and interferon/MHC-I activation as effect modifiers within a stratified or adaptive-enrichment analysis. Stage 1 markers can be evaluated more cheaply, on archival baseline tissue from completed trials including CALLA and KEYNOTE-A18, provided the analysis plan is registered in advance and the composite primability index is locked before unblinding rather than fitted to outcome. Three commitments separate biomarker qualification from description at every stage: prespecified thresholds, blinded assessment, and explicit reporting of incremental value over standard clinicopathological factors. We would regard the framework as refuted, and not merely unproven, if a study meeting these conditions found that on-treatment activation status and lymphocyte nadir did not modify the effect of checkpoint blockade.

## 9 Beyond checkpoint blockade: antigen-directed therapies and their timing

This temporal logic extends beyond PD-(L)1 antibodies. Any immunotherapy that acts through T cells follows the same four-state trajectory. Because cervical cancer carries defined viral antigens, it is a natural setting for strategies that supply or amplify HPV-specific immunity directly, instead of merely releasing a brake on an existing response. Reading these approaches along the treatment time axis clarifies both why several have shown activity and why combining them with CRT remains an open scheduling problem.

Adoptive cell therapy offers the clearest evidence that HPV-specific T cells can eradicate established disease. In a small pilot cohort of women with metastatic cervical cancer, a single infusion of HPV E6/E7-reactive tumor-infiltrating lymphocytes produced durable complete regressions in a subset of patients. Two features of that trial bear on the present argument: both the magnitude of HPV reactivity in the infusion product and the persistence of HPV-reactive T cells in peripheral blood one month after treatment correlated with clinical response (111). A subsequent phase II study confirmed objective responses in roughly a quarter of patients with cervical cancer, again tracking the HPV reactivity and blood repopulation of the transferred cells (112). For the CRT setting, these trials carry two implications. They first identify the effector population that matters, the viral-antigen-specific T cells, and show that its peripheral kinetics report on efficacy, which is exactly the readout the temporal biomarker framework (Section 8) proposes to monitor during CRT. They were also delivered after lymphodepleting conditioning, a deliberate reset of the compartment that CRT depletes inadvertently through radiation-induced lymphopenia (Section 4). The comparison shows that whether lymphodepletion helps or harms turns on a single factor: whether it is followed by reconstitution of an antigen-specific pool. Engineered T-cell therapies extend the same principle. HPV E7-directed TCR-T cells have established the treatment of HPV-associated cancers as a model for adoptive therapy of epithelial malignancies, offering an off-the-shelf route to the same viral-antigen-specific effector population (113).

Therapeutic vaccination aims to generate that population endogenously, and its clinical signal is strongest in combination with checkpoint blockade. In advanced HPV-positive cervical cancer, the GX-188E DNA vaccine given with pembrolizumab produced responses at roughly double the rate reported for pembrolizumab monotherapy, and responses were enriched among patients who mounted HPV-specific T-cell responses (114). The same combinatorial pattern held across HPV-16-driven cancers for the ISA101 synthetic long-peptide vaccine plus nivolumab, where the overall response rate exceeded that expected for the antibody alone and correlated with vaccine-induced T-cell activation (115). More recently, the HPV16 DNA vaccine VB10.16 combined with atezolizumab in persistent, recurrent, or metastatic cervical cancer was well tolerated and produced durable responses, with activity concentrated in tumors that were not heavily PD-L1-excluded (116). Across these studies, vaccination and checkpoint blockade act as complementary interventions: the vaccine supplies antigen-specific T cells and the antibody prevents their re-inhibition. In effect, the combination recapitulates *in vivo* the priming-and-release sequence that CRT itself attempts. The pattern reframes the negative and positive CRT–ICI trials of Section 7. A weakly priming regimen may fail for want of an antigen-specific pool to release, rather than because checkpoint blockade is inactive. Vaccination or cell transfer could supply that pool.

For these modalities, the timing problem returns in sharper form. CRT is, on the mechanistic reading of Section 3, an endogenous *in situ* vaccine, and direct human evidence now supports this in cervical cancer specifically: serial deep sequencing of the intratumoral T-cell receptor repertoire during chemoradiation with brachytherapy documented expansion of HPV E6/E7-responsive clones over the treatment course in a majority of patients, and, tellingly, the two patients who recurred were those who failed to remodel their repertoire at all (46). If CRT itself primes HPV-specific clones, then exogenous vaccination or cell transfer is best positioned either to amplify that endogenous response during the priming window or to reconstitute it afterward in patients whose own repertoire fails to remodel, exactly the failure mode that the on-treatment biomarkers of Section 8 are meant to detect. The corollary is that antigen-directed therapy and checkpoint blockade should be scheduled by the patient’s measured trajectory rather than by convention: augment priming where the endogenous response is weak, release the brake once a response has been generated, and reconstitute the pool where lymphodepletion has erased it. None of these combinations has been tested in the curative CRT setting with serial correlative sampling, which is the central evidence gap this review identifies for antigen-directed as much as for checkpoint-directed immunotherapy.

## 10 Conclusion and future directions

Cervical cancer offers an unusually complete opportunity to study immunotherapy in time: a defined viral antigen, a standard cytotoxic backbone that is itself an immune modulator, and now positive and negative phase III trials of added checkpoint blockade. Organizing the evidence along the treatment axis, rather than by cell type or by a single baseline snapshot, reframes the field’s central question. On the reading advanced here, and as a hypothesis rather than a demonstrated result, the determinant of CRT–ICI benefit may be not only whether a tumor is inflamed at baseline, but whether the patient completes the dynamic transition from viral-antigen exposure through antigen presentation and T-cell recruitment to durable post-treatment surveillance, and whether checkpoint blockade is present at the windows when that transition is most vulnerable.

Three priorities follow. First, prospective longitudinal sampling of paired tumor and blood at pre-, mid-, and post-treatment timepoints should become a standard correlative design for CRT–ICI trials, so that candidate explanations for trial heterogeneity can be tested as mechanism rather than inferred. Second, ctHPV-DNA-guided adaptive strategies (escalation for non-clearers, de-escalation or surveillance for rapid clearers) are ripe for formal evaluation. Third, the temporal biomarker framework should be validated and refined in cervical-specific cohorts, with attention to standardizing the separation of tumor-local and peripheral readouts. Cutting across all three, the treatment time axis reframes not only checkpoint blockade but the wider immunotherapy armamentarium. Therapeutic vaccination, adoptive cell therapy, and TCR-engineered T cells all act on the same antigen-specific effector population. They are therefore candidates to be scheduled by a patient’s measured trajectory rather than by convention (Section 9). The current evidence carries four main limitations. Much of the mechanism is preclinical or extrapolated from other tumor types. Human longitudinal datasets remain small and heterogeneous. The antigen-anchored components (ctHPV-DNA, viral-clone tracking) apply to HPV-associated disease and would need re-derivation for HPV-independent histologies, for which several baseline markers may also require an inverted reading (Section 5.1). And the proposed framework is conceptual and not yet prospectively validated, although Section 8 now specifies the study designs that would qualify or refute each of its three stages. A fifth limitation is that this review addresses efficacy far more thoroughly than toxicity. The phase-resolved, organ-specific safety data that would allow timing to be optimized jointly for benefit and harm are not currently published for either pivotal trial (Section 7.1). Addressing these gaps will determine whether the timing of immunotherapy in cervical cancer can be moved from trial-level empiricism to patient-level, biomarker-guided decision-making.
